# Variability of Inverted Repeats in All Available Genomes of Bacteria

**DOI:** 10.1128/spectrum.01648-23

**Published:** 2023-06-26

**Authors:** Otília Porubiaková, Jan Havlík, Indu, Michal Šedý, Veronika Přepechalová, Martin Bartas, Stefan Bidula, Jiří Šťastný, Miroslav Fojta, Václav Brázda

**Affiliations:** a Institute of Biophysics of the Czech Academy of Sciences, Brno, Czech Republic; b Brno University of Technology, Faculty of Chemistry, Brno, Czech Republic; c Department of Biology and Ecology, Faculty of Science, University of Ostrava, Ostrava, Czech Republic; d School of Pharmacy, University of East Anglia, Norwich Research Park, Norwich, United Kingdom; e Mendel University in Brno, Brno, Czech Republic; f Brno University of Technology, Faculty of Mechanical Engineering, Brno, Czech Republic; g Department of Experimental Biology, Faculty of Science, Masaryk University, Brno, Czech Republic; Connecticut Agricultural Experiment Station

**Keywords:** inverted repeats, Palindrome analyser, bacteria domain, bacterial genome analysis

## Abstract

Noncanonical secondary structures in nucleic acids have been studied intensively in recent years. Important biological roles of cruciform structures formed by inverted repeats (IRs) have been demonstrated in diverse organisms, including humans. Using Palindrome analyser, we analyzed IRs in all accessible bacterial genome sequences to determine their frequencies, lengths, and localizations. IR sequences were identified in all species, but their frequencies differed significantly across various evolutionary groups. We detected 242,373,717 IRs in all 1,565 bacterial genomes. The highest mean IR frequency was detected in the *Tenericutes* (61.89 IRs/kbp) and the lowest mean frequency was found in the *Alphaproteobacteria* (27.08 IRs/kbp). IRs were abundant near genes and around regulatory, tRNA, transfer-messenger RNA (tmRNA), and rRNA regions, pointing to the importance of IRs in such basic cellular processes as genome maintenance, DNA replication, and transcription. Moreover, we found that organisms with high IR frequencies were more likely to be endosymbiotic, antibiotic producing, or pathogenic. On the other hand, those with low IR frequencies were far more likely to be thermophilic. This first comprehensive analysis of IRs in all available bacterial genomes demonstrates their genomic ubiquity, nonrandom distribution, and enrichment in genomic regulatory regions.

**IMPORTANCE** Our manuscript reports for the first time a complete analysis of inverted repeats in all fully sequenced bacterial genomes. Thanks to the availability of unique computational resources, we were able to statistically evaluate the presence and localization of these important regulatory sequences in bacterial genomes. This work revealed a strong abundance of these sequences in regulatory regions and provides researchers with a valuable tool for their manipulation.

## INTRODUCTION

DNA molecules store genetic information for all cellular organisms. The arrangements of individual bases in the DNA sequences of an organism are specific, and elucidation of massive numbers of genome sequences has impacted our understanding of the phylogenetic tree of life (1). DNA molecules mostly form a double-stranded, right-handed helical B-form structure (2–4). However, DNA has been confirmed to form various alternative non-B structures (5). These structures include cruciforms (6, 7), Z-DNA (8), triplexes (9, 10), four way-DNA structure G-quadruplexes (G4s) and i-motifs (11–13), slip DNA (11), and sticky DNA structures (14, 15).

Bacterial genomes are mostly circular and usually consist of large chromosomes and small plasmids (16). In these complex cellular environments, various local DNA structures appear to be markers of specific activities or functions. Several studies have demonstrated the role of cruciform-forming IRs in the genomic replication of plasmids, mitochondrial DNA (17), and chloroplast DNA (18). They also play a role in dynamic genome organization (19), genomic stability, and transcription (20). Cruciform structures also play important roles in various diseases, such as cancer or Werner syndrome (6), and interact with various architectural and regulatory proteins, such as histone H1 (21), H5, topoisomerases, p53 (22), DEK proto-oncogene (23), and others (6).

**FIG 1:**
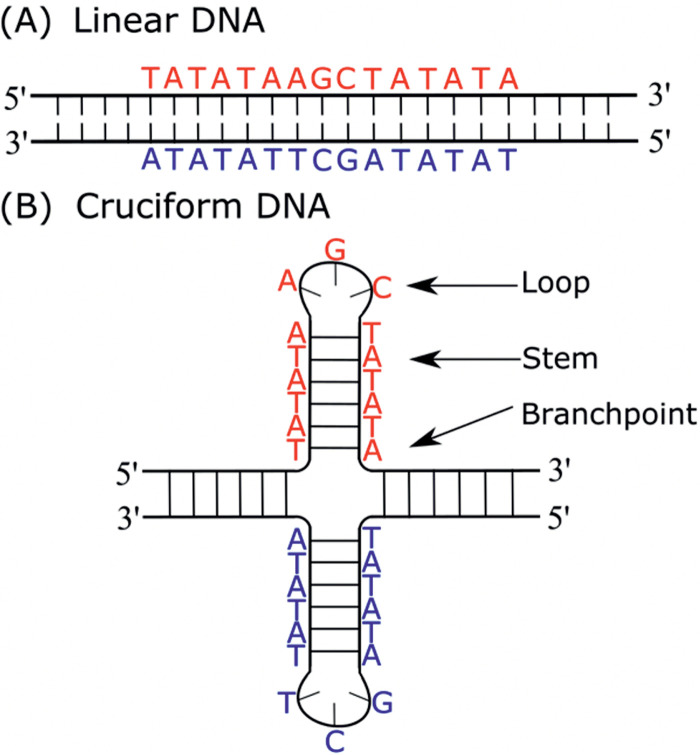
Inverted repeat in linear DNA (A) and in cruciform structure (B).

Cruciform structures consist of a branch point, a stem, and one or more loops. A loop size depends upon the length of the gap between inverted repeats (IRs) (Fig. 1). Direct IRs (without a gap in the repeat sequence), also called palindromes, lead to the formation of a cruciform with small loops. The cruciform formation in indirect IRs is dependent on the length of the repeat region and on the sequence in the gap (which forms the single-stranded loop in the assembled cruciform). Generally, the presence of AT sequences increases the probability of cruciform formation (6). Atomic force microscopy has been used to visualize cruciform geometry and revealed two classes of cruciform, as follows: unfolded, with a square planar conformation characterized by a 4-fold symmetry in which adjacent arms are nearly perpendicular to one another, and a folded conformation, in which the adjacent arms form an acute angle with the main DNA duplex (24). Holliday junctions, where two of the three structural motifs (4-strand, branch point, and double-stranded stem) are present, are structurally similar to cruciforms. These junctions are formed during recombination, fork reversal, and double-strand break repair during replication. They are resolved by junction-resolving enzymes, and this resolution is an essential process for maintaining genomic stability (25, 26).

Due to the roles of IRs in basic cellular processes, it is important to understand their presence, type, and localization in genomes. Although there are several tools developed for IR analyses, usually they are not user friendly and/or easily accessible or exhibit limitations in genome-wide analyses. For example, MFOLD can detect cruciform structures within an input sequence of up to only 9,000 bases (27). We have used our Web platform called Palindrome analyser, which allows IR analyses without size limitation and is suitable for circular genomes (28).

In the present study, we analyzed the presence and locations of IRs in 1,565 fully sequenced bacterial genomes using the Palindrome analyser. Our data show the distribution of IRs in individual phylogenetic groups and subgroups. Their specific localizations indicate the importance of IRs in regulatory processes within the domain *Bacteria*.

## RESULTS

**FIG 2:**
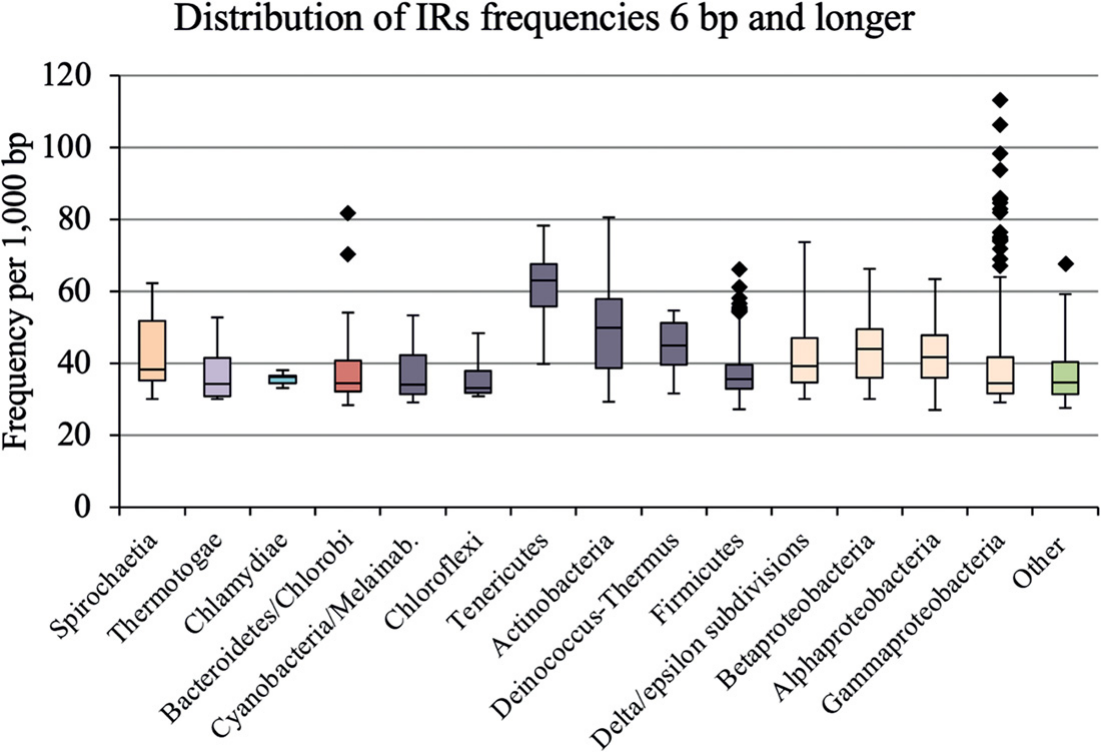
Frequencies of IRs in subgroups of the analyzed bacterial genomes. Data within boxes span the interquartile range, and whiskers show the lowest and highest values within the 1.5 interquartile range. Black diamonds denote outliers (Table S7).

All fully assembled genomes from the domain *Bacteria* were downloaded from the NCBI database (4 Sep 2020). In total, we analyzed 1,565 bacterial genomes for the presence of IRs by using Palindrome analyser with the default parameters (length of repeat, 6 bp and more; length of gap, 0 to 10 bp; 0 or 1 mismatch allowed). The data were then sorted according to NCBI taxonomy classifications into 18 groups and 39 subgroups. For the statistical evaluation, only groups of 10 or more species with sequenced genomes were used. The lengths of bacterial genomes in the data set ranged from 200 kbp (Buchnera aphidicola and Acyrthosiphon kondoi) to 13 Mbp (Sorangium cellulosum). The average GC content was 54.15%, with a minimum of 20.10% for *B. aphidicola* (subgroup *Gammaproteobacteria*) and a maximum of 80.52% for Corynebacterium sphenisci (subgroup *Actinobacteria*). The basic statistical parameters for all genomes as well as those for individual groups and subgroups are shown in Table 1. The total number of nucleotides in the 1,565 bacterial genomes analyzed was 5,776,630,336, where 242,373,717 IRs were found with a mean frequency of 41.88 IRs per 1,000 bp. For most organisms, IR frequencies were found in the range of 30 to 80 IRs/kbp (Table 1; Fig. 2). However, 11 organisms had IR frequencies exceeding 80 IRs/kbp. With the exception of one genome from *Terrabacteria*, all of them belonged to *Proteobacteria*. The highest frequency of 113.37 IRs/kbp was found in *B. aphidicola*. The lowest IR frequency (27.08 IRs/kbp) was found for Anaplasma centrale belonging to the *Alphaproteobacteria* subgroup. The highest mean frequencies per kbp were found for the *Terrabacteria* (44.07) and *Spirochaetes* (43.14), followed by the *Proteobacteria* (41.32). The lowest mean IR frequencies were found in the *Thermotogae* (37.06) and the *Planctomycetes*, *Verrucomicrobia*, *Chlamydiae* (PVC) groups (35.66). By an analysis of domain *Bacteria* subgroups, the highest frequency of IRs/kbp was observed for the *Tenericutes* subgroup (61.89) and the lowest one was observed for the *Chloroflexi* subgroup (34.98). Detailed statistical comparisons are available in Table S3 (groups) and Table S4 (subgroups) in the supplemental material.

**TABLE 1:** IR characteristics in bacterial genomes^*a*^

Bacteria	Seq	Median	Short	Long	IRs	Mean f	Min f	Max f	GC%
Overall	1,565	3,669,183	200,073	13,033,779	242,373,717	41.88	27.08	113.37	54.15
Group									
*Spirochaetes*	33	2,889,325	900,755	4,653,970	3,490,999	43.52	30.06	62.24	42.95
*Thermotogae*	16	2,150,379	1,884,562	2,974,229	1,266,797	37.06	30.12	52.72	39.27
PVC group	12	1,168,953	1,041,170	3,072,383	688,281	35.66	33.18	38.08	40.66
FCB group	112	3,954,701	605,745	9,127,347	16,875,955	38.09	28.52	81.83	42.29
*Terrabacteria*	647	3,051,613	564,395	11,936,683	1,07,237,517	44.07	27.74	80.52	54.86
*Proteobacteria*	656	3,899,679	200,0.73	13,033,779	102,827,992	41.32	27.08	113.37	56.58
Other	89	2,476,671	1,125,857	9,629,675	9,986,176	36.66	27.72	67.76	51.89
Subgroup									
*Spirochaetia*	33	2,889,325	900,755	4,653,970	349,099	43.53	30.06	62.24	42.95
*Thermotogae*	16	2,150,379	1,884,562	2,974,229	1,266,797	37.06	30.06	52.72	39.27
*Chlamydiae*	12	1,168,953	1,0141,170	3,072,383	688,281	35.66	33.18	38.08	40.66
*Bacteroidetes/Chlorobi*	112	3,954,701	605,745	9,127,347	16,875,955	37.51	28.52	81.83	42.29
*Cyanobacteria/Melainab.*	29	5,315,554	1,657,990	9,673,108	5,105,883	37.57	29.28	53.44	43.35
*Chloroflexi*	11	2,574,431	1,362,151	5,723,298	1,222,098	34.98	30.88	48.15	57.55
*Tenericutes*	52	981,001	564,395	1,877,792	3,250,026	61.89	39.82	78.29	28.04
*Actinobacteria*	245	3,973,750	927,303	11,936,683	59,613,628	49.21	29.30	80.52	68.05
*Deinococcus-Thermus*	18	2,895,912	2,035,182	3,881,839	2,265,314	45.21	31.71	54.71	66.68
*Firmicutes*	292	2,936,195	1,274,073	11,456,784	34,338,540	37.50	27.24	65.93	41.39
Delta/epsilon subdivisions	92	3,136,746	1,457,619	13,033,779	14,578,664	41.92	30.20	73.82	55.73
*Alphaproteobacteria*	194	3,725,037	859,006	9,207,384	29,277,488	42.42	27.08	95.30	61.28
*Betaproteobacteria*	96	4,171,754	820,037	9,731,138	17,943,881	42.67	30.05	66.39	62.06
*Gammaproteobacteria*	274	4,089,965	200,073	9,336,592	34,320,721	39.86	29.16	113.37	51.90
Other	89	2,476,671	1,125,857	9,629,675	9,986,176	36.66	27.72	67.76	51.89

a Seq, no. of sequences in the data set; median, median length of sequences; short, shortest sequence; long, longest sequence; IRs, total no. of predicted IRs; mean f, mean frequency of predicted IRs per 1,000 bp; min f/max f, highest/lowest frequency of predicted IRs per 1,000 bp; GC%, average GC content (%) (from Table S2, S3, and S4).

The Shapiro-Wilk test of IR frequencies showed that the data were not normally distributed (W = 0.87 and *P* = 0), and the Kruskal–Wallis signed-rank test indicated that IR frequencies in bacterial DNA differed significantly (*P < *0.05) (available in Table S8 in the supplemental material). A graphical representation of the IR frequencies is shown in Fig. 2.

**FIG 3:**
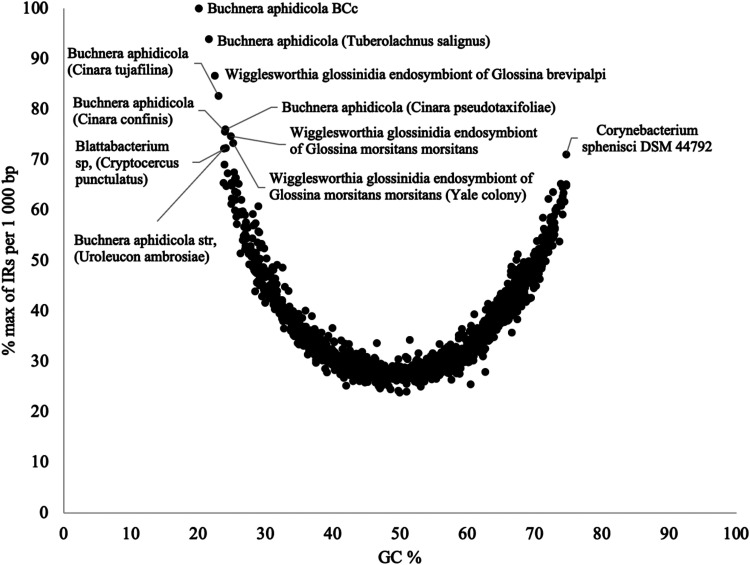
Relationship between observed IR frequencies per 1,000 bp and GC content in all analyzed bacterial genomes. Frequencies were normalized according to the highest observed frequency of IRs, and organisms with maximal frequency per 1,000 bp greater than 70% were described (Table S5).

We visualized the relationship between the content of GC (%) in the genomes and the frequency of IRs (Fig. 3; see Table S5 in the supplemental material). Organisms with high IR frequencies relative to their GC content (over 70% of the maximal observed IR frequency) were identified. Almost all 11 outliers belonged to *Proteobacteria*, except *Blattabacterium* (Cryptocercus punctulatus; *Fibrobacterota*, *Chlorobiota*, and *Bacteroidota* [FCB] group) and *C. sphenisci* (*Terrabacteria*). All 9 outliers from the *Proteobacteria* group belonged to the *Gammaproteobacteria* subgroup, within which is the organism with the highest IR frequency (113.37 IRs/kbp, *B. aphidicola*). Our results were in accordance with the previous findings on the relationship between the frequency of palindromes and GC content (29), when the survey was conducted on a smaller data set.

**FIG 4:**
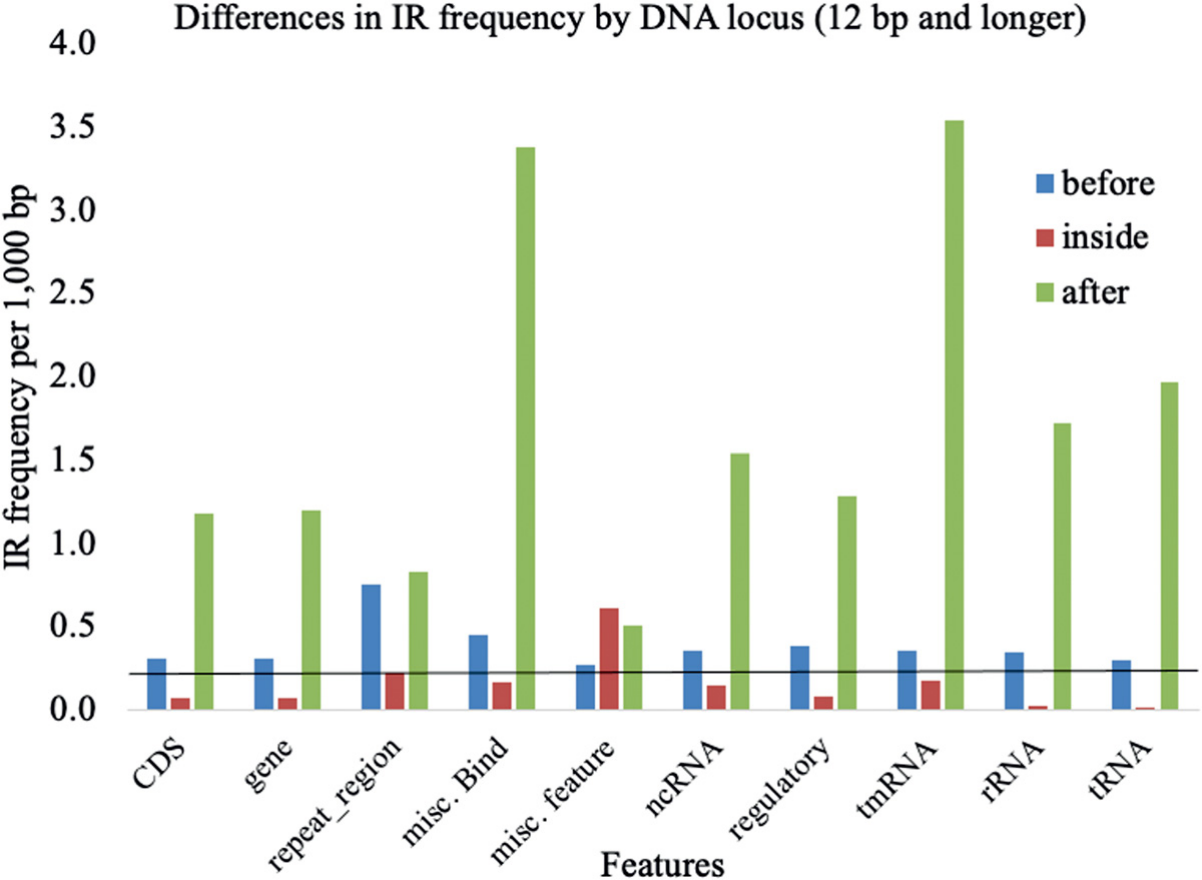
Differences in IR frequency by DNA locus. IR that were 12 bp and longer within annotated locations and 100 bp before or after annotated locations were analyzed (all data in Table S6). The line indicates the mean frequency for IRs of 12 bp and longer.

Next, we downloaded the annotated features of all bacterial genomes and identified the genomic location of all IRs. Among those described, IRs could be found in the coding regions (CDSs), genes, repeat regions, miscellaneous binding (misc_bind), miscellaneous features (misc_feature), noncoding RNAs (ncRNAs), regulatory domains, sequence-tagged sites (STSs), transfer-messenger RNAs (tmRNAs), rRNAs, and tRNAs (Fig. 4; see Table S6 in the supplemental material). For a comparison of IR frequencies at different locations, the most common annotation, “gene,” was used as a standard. Significant differences in IR frequencies were found in various features of DNA.

We found that the IR coverage decreased with increasing IR length, and we found differences in IR distribution. The greatest frequency of IRs longer than 12 bp was found within the “miscellaneous features.” However, the most notable and significant enrichment of IRs was found predominantly before and after the annotated features. This finding applied to genes and RNAs, thus indicating a potential crucial regulatory role of IRs. For IRs of size 12+ bp, significant enrichment was found inside the tmRNAs and repeat regions. In particular, the longer IRs showed a nonrandom distribution in the bacterial genomes and were found in greater abundance around annotated features. Because the presence of IRs leads to genome plasticity, organisms with a higher frequency of IRs have more dynamic genomes that additionally contains genes encoding, for example, resistance, antibiotic production, or pathogenicity (30). All data for coverage and IRs ratios in various features are provided in Table S6.

In the following text, eight selected examples of bacterial species with extraordinary IR patterns are described. The absolutely highest genomic GC content was found in Corynebacterium sphenisci, which is a Gram-positive bacterium. This species consists of nonmotile, non-spore-forming rods and is facultatively anaerobic. The incubation period is 48 h at 37°C on sheep blood agar (31). Overall, IR frequency per 1,000 bp was equal to 80.52 and the highest relative enrichment of IR frequencies was after ncRNA (125.00) and after regulatory (123.75) features. The absolutely lowest GC content was found in the Buchnera aphidicola genome. This bacterium is an endosymbiont of aphids, has a genome size of 600 to 650 kb (which encodes on the order of 500 to 560 proteins), and also transmits viruses with the help of a symbionin protein. Buchnera aphidicola is found in bacteriocytes in most of the 4,400 aphid species, supplying the aphids with essential amino acids. In return, Buchnera aphidicola is given a stable and nutrient-rich environment. This aphid-*Buchnera* relationship has existed since 250 million years ago (32). Interestingly, this species has also the highest IR frequency per 1,000 bp equal to 113.37, and the highest relative enrichment of IR frequencies was after CDSs (200.22), after tmRNAs (200.00), after gene features (194.76), and before CDS regions (180.22).

Considering species with a GC content of around 30%, two species with mutually contrasting IR patterns were selected. Borrelia anserina has a GC content of 29.49% and an overall genomic IR frequency per 1,000 bp equal to 48.61. There was no exceptional enrichment of IR frequencies considering particular genomic features. Borrelia anserina is the cause of chicken spirochaetosis and is spread around the world by ticks of the genus *Argas*. The relapsing fever phenotype of Borrelia anserina sets it apart from other *Borrelia* species. The genome consists of a megaplasmid and a linear chromosome that is around 900 kb long. Although it has been discovered that Borrelia anserina can be grown on Barbour-Stoenner-Kelly (BSK) medium for a superior yield, it has traditionally been kept in embryonated chicken eggs (33). The contrast species, a secondary endosymbiont of Heteropsylla cubana belonging to the *Pseudomonadota Gammaproteobacteria* group (34), had a GC content of 28.90% and an overall genomic IR frequency per 1,000 bp equal to 68.87. Marked relative enrichment of IR frequencies was found before (125.00) and after (135.00) ncRNA features.

Considering species with a GC content of around 50%, two species with contrast IR patterns were selected as well. Treponema brennaborense DSM 12168 was isolated from a dairy cow suffering from digital dermatitis. Treponema is a genus of Gram-negative spirochetes, which are characterized by their distinctive, spiral shape. Some species of Treponema have been associated with human and animal diseases, such as syphilis and periodontitis, but Treponema brennaborense DSM 12168 is not known to cause any harm to humans (35). This species had a GC content of 51.47 and an overall genomic IR frequency per 1,000 bp equal to 38.84. The highest relative IR frequency was found inside the repeat regions’ genomic feature (53.14). The contrast species, Akkermansia glycaniphila of the strain PytT, is a Gram-negative, nonmotile, and anaerobic bacteria. This mucin-degrading bacteria was identified initially in the reticulated python’s intestine (36). Mucin gives microorganisms nitrogen and carbon. The strain PytT genome was 3.07 Mbp in size and a GC content of 57.7% (37). The overall IR frequency per 1,000 bp for this species was equal to 29.90. The highest relative enrichment of IR frequency in this species was found after tmRNA features (70.00).

Finally, considering species with a GC content of around 70%, two species with contrast IR patterns were selected. Burkholderia ubonensis is a nonpathogenic soil bacterium that is a part of the Burkholderia cepacia complex (Bcc), a collection of genetically connected organisms linked to opportunistic, usually nonfatal infections in healthy people. This species with a GC content of 67.50% had an overall IR content per 1,000 bp equal to 51.29. The highest relative enrichment of IR frequency was found inside regulatory features (67.58). Burkholderia ubonensis has the potential to be a significant biocontrol agent for Burkholderia pseudomallei due to the fact that some strains are hostile to it. Burkholderia pseudomallei causes melioidosis, a condition that, if untreated, can be fatal in up to 95% of cases (38). The contrast species, Myxococcus xanthus, is a Gram-negative soil bacterium belonging to the delta subgroup of proteobacteria, having a genome size of 9.14 Mb. An estimated 8% of the Myxococcus xanthus genome is dedicated to the production of secondary metabolites, and at least 18 gene clusters specify the production of polyketide which is a model for antibiotic production (39). This species with a GC content of 68.89% had an overall IR content per 1,000 bp equal to 42.71. The highest relative enrichment of IR frequency was found after rRNA features (56.67).

**FIG 5:**
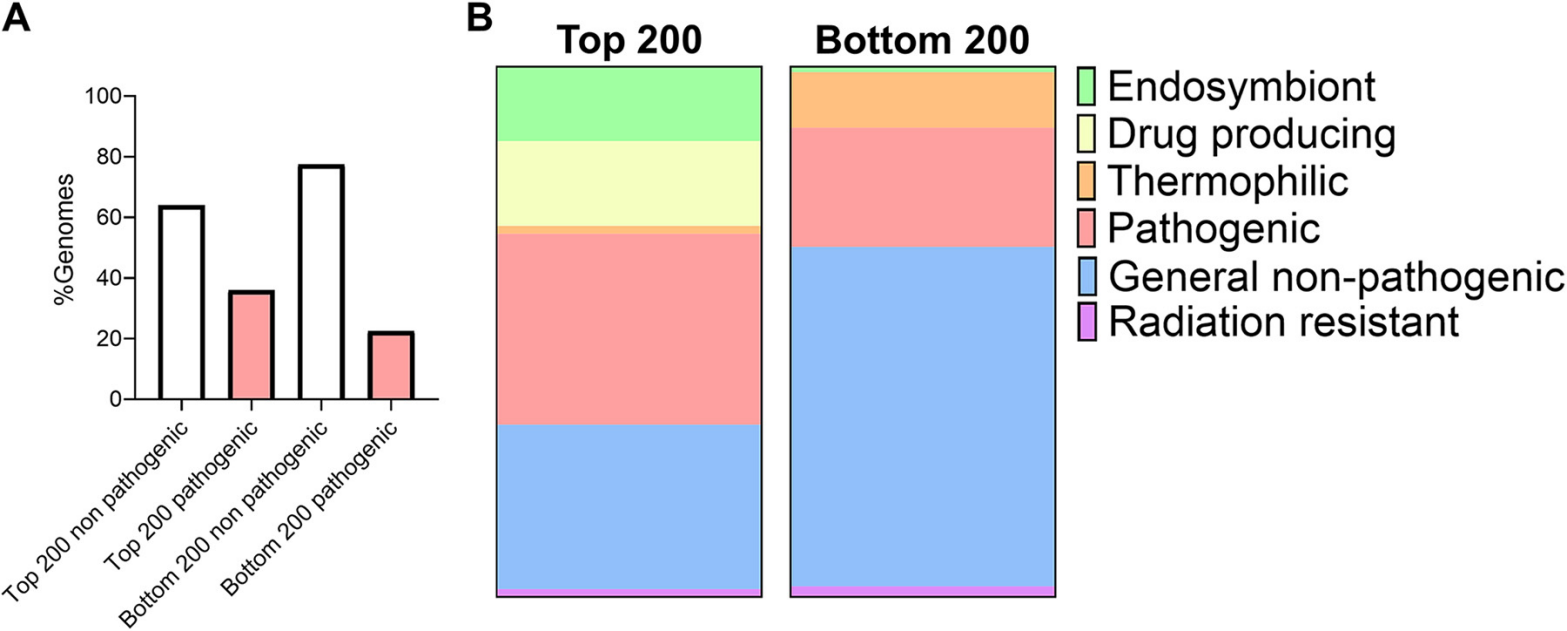
Organisms with higher IR frequencies are more likely to be pathogenic, to be endosymbiotic, or produce antibiotics. (A) The percentage of organisms that were pathogenic or nonpathogenic among those with the highest or lowest IR frequencies. (B) The 200 organisms with the highest and 200 with the lowest genome IR frequencies were further subdivided into organisms that were endosymbionts, drug producing, thermophilic, pathogenic, radiation resistant, or generally nonpathogenic (Table S8).

We then explored a possible association between the frequency of IRs and pathogenic potential. To investigate this association, we compared 200 genomes with the highest IR frequencies to 200 genomes with the lowest IRs frequencies and noted whether these organisms had been reported previously as pathogenic. We found that pathogenic bacteria were more likely to have higher genome IR frequencies, with 72 pathogenic bacteria listed in the top 200 (36%) compared with 45 (22.5%) in the bottom 200 (Fig. 5A; see Table S7 in the supplemental material). These species were further separated into endosymbionts, namely, those which produce antibiotics/clinically relevant drugs, thermophiles, radiation-resistant, pathogenic, or generally nonpathogenic (e.g., including species that fix nitrogen). Bacteria with a higher genome frequency of IRs were found to be more likely pathogenic, endosymbiotic, or involved in the production of antibiotics than bacteria with a low genome frequency of IRs (Fig. 5).

This finding highlighted that bacteria with higher genome IR frequencies were more likely to be endosymbiotic (28/200, 14%), involved in the generation of antibiotics (32/200, 16%), or pathogenic (72/200, 36%) than those with lower IRs genome frequencies (1%, 0%, and 22.5% of the bottom 200 genomes, respectively) (Fig. 5B). Conversely, bacteria with lower IR frequencies were more likely to be thermophilic (21/200, 10.5%) and generally nonpathogenic (128/200, 64%) than bacteria with higher IR genome frequencies (1.5% and 31% of the top 200 genomes, respectively) (Fig. 5B).

## DISCUSSION

DNA cruciforms play important roles in transcription regulation by interacting with various proteins, such as helicases, PARP-1, BRCA1, p53, and many others (6, 40, 41). Today’s bioinformatic tools allow analyses of complete genomes and bring a more complete view of DNA structure and regulation. Here, using the Palindrome analyser, we analyzed all complete bacterial genomes available from the NCBI (1,565) for the presence and localization of IRs. We identified IRs of lengths ranging from 6 to 30 bp having the ability to form cruciform structures. While the mean frequency of IRs was 41.78 IR/kbp, the particular frequencies were notably higher in some specific subgroups (*Tenericutes* and *Actinobacteria*). The highest mean frequency was noted for the *Tenericutes* subgroup (61.89), with 3,250,026 IRs found and low GC content (28.04%). A previous analysis of putative G-quadruplex-forming sequences (PQSs) had shown that the same subgroup had the lowest PQS frequency. An inverse relationship between the frequency of PQSs and IRs was also observed recently in mitochondrial genomes and is not unique to the *Tenericutes* (17, 42). On the other hand, the *Actinobacteria* subgroup had a high GC content of 68.08%, a high frequency of IRs, and also a high frequency of PQSs (43). The highest IR frequencies (>70 IRs per 1,000 bp) were found in *B. aphidicola*, Wigglesworthia glossinidia, *Blattabacterium* sp., and *C. sphenisci*. The very highest IR frequency was present in *B. aphidicola* BCc (113.37 per kbp), which has a genome size of only 416,380 bp. *Buchnera* spp. are minuscule endosymbiotic bacteria, and their genomes encode only around 500 proteins. One of the lowest mean IR frequencies was found in the *Chlamydiae* subgroup (35.66), with 688,281 identified IRs. This subgroup includes obligate intracellular parasites (44). The overall lowest number of IRs was found in Butyrivibrio hungatei from the *Terrabacteria* group (34,609 IRs). The presence of IRs in replication origins and other regulatory regions is known from previous analyses (18, 45, 46), and it has been demonstrated that hairpins formed in the IRs can regulate RNA polymerases (47). Our analyses showed that short IRs are nonrandomly distributed and that most IRs are located around annotated features rather than within annotated features. It has been shown that long IRs (12 bp and longer) are associated with amplified genes, and in humans, it has been suggested that they are important in late tumor progression (48). Their enrichment was found inside the tmRNA that participates in the rescue process in the case that the ribosomes cannot finish translation (49). Our analyses showed the highest IR coverage also inside miscellaneous binding, regulatory, tRNA, and ncRNA features. The category miscellaneous feature is general and can encompass a wide range of biologically important sequences. According to the NCBI, it is a region of biological interest that cannot be described by any other feature; potentially it includes new or rare features (50). Most of the long IRs in miscellaneous features were associated with rRNAs. Particularly interesting was their presence in the apical loop-internal loop (ALIL) category, which is associated with frameshifting in bacteria and serves to modulate the expression of minority genes. Here, the presence of secondary structures plays an important role, particularly if the structure is located at the 3′ side of the shift site, where it serves as barrier to mRNA translocation and causes ribosome pausing (51). Our analyses revealed the presence of numerous IRs across all available bacterial genomes. These repeats have important consequences for genome stability, but they could also be under positive selection for antigenic variation. Thus, they appear to exist at a juncture where the need to generate genetic diversity coincides with a need to limit that diversity (52). Present studies suggest that the presence of a long IR near the replication terminus can be helpful for chromosome rescue after premature replication termination or irreversible chromosome damage (53).

Finally, we also found that pathogenic bacteria were slightly more likely to have a higher frequency of IRs in their genomes. This observation has been extended recently to viral pathogens, as the gene encoding the SARS-CoV-2 spike protein and the SARS-CoV-2 genome itself is particularly enriched with IRs (54, 55). As IRs are frequently found located within mutation hot spots, and mutation has enhanced the propagation of the virus, it could be hypothesized that an increased genome frequency of IRs may also provide a survival advantage to the bacterial pathogens. Therefore, IRs may also play an important but underappreciated role in the pathogenesis of microorganisms.

In conclusion, here, we analyzed the presence of IRs in 1,565 bacterial genomes using the Palindrome analyser. We described basic parameters, including the frequency and localization of IRs and their ability to form cruciforms. IRs were identified in all examined species with notable differences between individual groups and subgroups. IRs were not located randomly, and IRs of sizes 12 bp or longer were enriched in specific genomic locations. The highest IR frequencies were found around the functional regions. Additionally, higher IR genome frequencies may be associated with pathogenicity, antibiotic production, and endosymbiosis. These data showed the nonrandom localization of IRs in bacterial genomes and their potential importance in basic and specialized biological processes.

## MATERIALS AND METHODS

### Analysis of IR frequency with Palindrome analyser.

All known bacterial genomes were downloaded in FASTA format from the genome database of the National Center for Biotechnology Information (NCBI) (56). We used only completely assembled genomes for our analysis and selected one representative genome for each species (see Table S1 in the supplemental material). The genomes were examined using Palindrome analyser (28) to detect the presence and localization of IRs. The parameters used for the IR analysis were an IR size of 6 to 30 bp, a spacer size from 0 to 10 bp, and a maximum of one mismatch. Subsequently, we created a separate list of IRs found in each bacterial genome. The overall results contained a list of species along with the sizes of their genomes, the numbers of IRs found in each sequence, and the frequency of the IRs (Table S2, S3, and S4). Table S5 includes the relationship between observed frequencies of IRs/bp and GC content in all analyzed bacterial genomes. Frequencies were normalized according to the highest observed frequency of IRs, and organisms with maximal frequency per 1,000 bp greater than 70% were described.

### Analysis of IRs around annotated NCBI features.

We downloaded tables containing the NCBI feature annotations and quantified the occurrence of IRs inside and around recorded features. From this analysis, we obtained a file including the feature names, coverage, and number of IRs found inside features for the analyzed domain. We quantified the amounts of all IRs and noted those longer than 8, 10, and 12 bp found inside features. Further processing was performed in Microsoft Excel (Microsoft 365, version 16.56), and all data are available in Table S6.

### Selection of eight representative bacterial species for in-depth qualitative analysis.

Corynebacterium sphenisci (GC content of 74.73%) and Buchnera aphidicola *BCc_chromosome_1* (GC content of 20.10%) were chosen due to their highest and lowest GC content, respectively. Then, two species with the similar GC contents close to GC contents of 30%, 50%, and 70% and at the same time to have different/opposite IR frequencies (one species low and the second high) were selected (GC content of each in brackets), as follows: *secondary endosymbiont of Heteropsylla cubana_chromosome_1* (28.9%), Borrelia anserina *Es_chromosome_1* (29.49%), Treponema brennaborense *DSM 12168* (51.47%), Akkermansia glycaniphila*_chromosome_1* (57.65%), Burkholderia ubonensis *MSMB22_chromosome_1* (67.5%), and Myxococcus xanthus *DK 1622_chromosome_1* (68.89%).

### Association between the frequency of IRs and potential pathogenicity.

Four hundred organisms were further analyzed (200 organisms with the highest and 200 with the lowest IRs frequencies) to determine whether they had previously been reported as pathogenic to humans, animals, or plants. We also noted whether organisms were antibiotic producing, endosymbionts, radiation resistant, thermophilic, or general nonpathogens. Data are available in Table S7.

### Statistical evaluation.

Statistical evaluation of normality was made using the Shapiro-Wilk test. Because the data were not normally distributed, we also analyzed normality using the Kruskal-Wallis signed-rank test to evaluate significant differences between groups and subgroups. A *post hoc* multiple pairwise comparison was performed using Dunn’s test with Bonferroni correction. Significance was determined where the *P* value was ≤0.05. Data are available in Table S8.

### Data availability.

The data sets supporting the conclusions of this article are available in the supplemental material.
